# A77 COMPLICATIONS OF DUODENAL DIVERTICULA: A SYSTEMATIC REVIEW ON CLINICAL PRESENTATION AND MANAGEMENT OF ENTEROLITH ILEUS SECONDARY TO DUODENAL DIVERTICULA FORMATION

**DOI:** 10.1093/jcag/gwad061.077

**Published:** 2024-02-14

**Authors:** C J Miranda, A M Carlson, N Hossein-Javaheri, A Aijaz, M H Ali, M Ismail

**Affiliations:** University at Buffalo, Buffalo, NY; University at Buffalo, Buffalo, NY; Internal Medicine, University at Buffalo, Buffalo, NY; University at Buffalo, Buffalo, NY; University at Buffalo, Buffalo, NY; University at Buffalo, Buffalo, NY

## Abstract

**Background:**

Duodenal diverticula (DD) are relatively common findings in the gastrointestinal (GI) tract with their presence noted in up to 20% of the population. Rarely, however, these diverticula predispose to enterolith formation which can lead to small bowel obstruction, a phenomenon termed “enterolith ileus”. Both surgical and non-surgical approaches have been espoused in tackling this complication but definitive literature on optimal management is lacking and is limited to scattered case reports and case series. With this phenomenon carrying significant morbidity and mortality, we have conducted a comprehensive review of all available case reports to summarize our understanding of this condition.

**Aims:**

To investigate the prevalance and clinical presentation of enterolith ileus secondary to duodenal diverticula and their optimal management whether that be medical or surgical.

**Methods:**

A comprehensive search of PubMed, OVID, CINAHL, and Cochrane databases up to February 2023 was conducted to identify all studies reporting clinical information on enterolith formation in DD leading to small bowel obstruction. Each article was qualitatively assessed, and relevant data were extracted from selected studies to determine clinical courses and optimal management.

**Results:**

Our literature review identified 17 case reports of enterolith ileus secondary to DD. The mean age of the patients was 72 years (SD 11.43), with 59% of them being female. Three patients had previously undergone Roux-en-Y gastric bypass, while one patient had undergone a distal gastrectomy. The most common symptoms reported were abdominal pain, nausea, and vomiting (88%), followed by abdominal distension (47%). Dilated bowel loops was the most common finding on X-rays, while CT scans revealed signs of small bowel obstruction and DD with an endoluminal mass in most of the cases. Upper GI series was performed in five cases, revealing multiple duodenal diverticula. Despite initial conservative management, 16 out of 17 patients eventually required surgical intervention. Enteroliths were extracted via enterotomy in 14 out of 17 cases, while surgical resection of the bowel was performed in three cases due to suspicion of tumor or perforation.

**Conclusions:**

Our findings highlight the importance of prompt diagnosis and management of DD leading to enterolith ileus, particularly in elderly patients and those with a history of gastric surgery. Surgical intervention is almost always required and therefore early recognition and intervention can help minimize the risk of morbidity and mortality associated with this condition.

**Funding Agencies:**

None

